# Evaluation of Circulating Leptin and Its Receptor (Ob-R) Tissue Expression in Colorectal Cancer, a Report From North of Iran

**DOI:** 10.30699/IJP.2023.1983482.3041

**Published:** 2023-07-16

**Authors:** Masoume Mahmoudi-Nesheli, Reza Alizadeh-Navaei, Laleh Vahedi, Omolbanin Amjadi, Tarang Taghvaei, Iradj Maleki, Ramin Shekarriz, Arash Kazemi, Versa Omrani-Nava, Maryam Alizadeh-Foroutan

**Affiliations:** 1 *Gastrointestinal Cancer Research Center, Non-Communicable Diseases Institute, Mazandaran University of Medical Sciences, Sari, Iran*; 2 *Department of Pathology, School of Medicine, Mazandaran University of Medical Sciences, Sari, Iran*; 3 *Gut and Liver Research Center, Department of Gastroenterology, Imam Hospital, Mazandaran University of Medical Sciences, Sari, Iran*

**Keywords:** Colorectal cancer, Immunohistochemistry, Leptin, Leptin receptor, LEP, LEPR, Phenotype

## Abstract

**Background & Objective:**

Leptin is an adipocyte-derived hormone with a critical role in energy balance. As demonstrated by previous investigations, leptin acts as a proliferative and angiogenic factor in cancer cells. However, results regarding its role in colorectal cancer are still inconclusive. We aimed to evaluate serum leptin and tissue expression of leptin receptor (Ob-R) in normal and malignant samples of colorectal.

**Methods:**

Serum and tissue samples from pathology-confirmed colorectal cancer patients and normal controls referring to a university hospital of Mazandaran were obtained during 2019-21. ELISA and immunohistochemistry were applied to determine leptin and Ob-R expression respectively.

**Results:**

A total of 90 samples belonging to 46 normal and 44 CRC patients were enrolled. Normal and CRC groups included 32 (69.56%) and 21 (47.72%) female subjects respectively. The average leptin concentration in the normal group was 115.80 and, in the patient, group was 124.47 ng/mL (*P*=0.897). CRC cases showed an insignificantly higher Ob-R detection rate (*P*=0.086).

**Conclusion:**

There was no significant difference in leptin and Ob-R expression between CRC patients and normal subjects. Thus, leptin and its receptor may not be useful as a biomarker of CRC.

## Introduction

Leptin is a peptide hormone with 146 amino acids which is mainly produced and secreted by fat cells of adipocyte tissues (1). The Leptin gene is located on chromosome 7 (7q31) and was first introduced in 1994 as the product of the obese gene. Studies declare that homozygous mutation in this gene results in mice obesity and infertility (2, 3). Leptin is an essential hormone in regulating food intake and energy balance. It exhibits wound-healing capacities by triggering epidermal keratinocytes proliferation, differentiation, and migration (4). Leptin membrane receptor (Ob-R) which is expressed mainly in the central nervous system belongs to the cytokine receptor family and comes with six different isoforms (5). Upon binding, Ob-R activates Janus kinase 2 (JAK2)/signal transduction and activator of transcription 3 (STAT3) signaling pathway which is critical in mediating immunological responses, as well as cellular proliferation and differentiation (6). Ob-R is not tissue-specific and has a wide tissue distribution which highlights its various functions. 

Besides their role in the physiologic conditions, leptin and Ob-R aberrant expression are linked to some pathologic conditions, including immune system-related diseases and malignancies. Cancer, as a challenging health issue, is a leading cause of mortality in many countries. Malignancies of the colon and rectum consisted of about 6% of new cancer cases and 576,858 oncological deaths in 2020 (7). Despite the highest colorectal cancer (CRC) incidence reported from developed countries, Iran is facing an increase in its trends (8). As the majority of CRC cases are sporadic environmental factors are said to play a crucial role in the development of this cancer. Therefore lifestyle-related factors like diet and physical activity can influence CRC risk (9). *A large body of evidence suggests* that substantially increased CRC risk is associated with obesity (10). Leptin is associated with obesity, therefore several effort has been *dedicated* to the role of leptin in CRC development (11). Leptin induces the proliferation of breast (12), colon (13), and gastric (14) cancer cells. Leptin influences the expression of some genes which are involved in colon cancer development (15). However, there are conflicts considering Leptin function and levels in CRC. Kumor *et al.* showed that serum Leptin in subjects without colorectal pathology is higher than in patients with adenoma or adenocarcinoma (16). Leptin causes overexpression of miR-4443 which is ob-R dependent and decreases CRC cell proliferation (17). According to Aparicio and colleagues, Leptin is a proliferative factor for different colon cancer cells in vitro nonetheless, hyperleptinaemia in the animal models did not contribute to tumorigenesis (13). In contrast, other studies claim Leptin is an important factor for colon tumor growth (18).

 Considering contradictory reports regarding the association between leptin / Ob-R levels and CRC (19), the present study aimed to evaluate serum leptin and tissue Ob-R expression in colonic mucosa obtained from cancerous and non-cancerous samples. 

**Selection of Patients**

After obtaining ethical approval, patients who were referred to Imam Hospital (Sari, Iran) during 2019-2021 were divided into colorectal adenocarcinoma and normal colonic mucosa according to the pathologic findings on biopsy samples from colonoscopic examinations. The age criterion for admission was ≥18 and the exclusion criteria were diabetes, hypertension, and hyperlipidemia. Anthropometric indices including BMI and fat percentage were measured by InBody 270 body composition analyzer. From each participant serum and tissue samples were used for ELISA and immunohistochemistry staining respectively prior to any surgery or chemotherapy. 

**Serum leptin Measurement**

Human leptin ELISA Kit (ZellBio GmbH, Germany) was applied according to instructions. About 5 mL of venous blood samples were collected from patients and serum was separated and stored at -20 for further analysis. For ELISA briefly, wells were coated with anti-leptin monoclonal antibodies. After adding a serum and binding the antibody to leptin, a biotin-conjugated anti-leptin antibody was added. In the next step, enzyme-linked streptavidin was added to bind to biotin. Color development was achieved by adding a substrate solution.

**Immunohistochemistry**

Paraffin-embedded samples used to diagnose CRC were applied for immunohistochemical staining. Briefly, 20 selected blocks were cut into 4-5 micrometer sections slices and were placed on the slide. In order to eliminate paraffin, slides were immersed in Xylene and ethanol. Citrate buffer was applied as a retrieval medium. Hydrogen Peroxide was added for blocking endogenous peroxidase activity. Then the slides were incubated with Anti-leptin receptor-antibody (Ob-R ZellBio GmbH) at a dilution of 1:200. Ob-R expression was analyzed by light microscopy in 10 different fields from each section and the mean percentage of cells that showed positive staining was scored by the pathologist. Staining intensities were calculated as 0, 1, 2, and 3, respectively, indicating negative, weak, medium, and strong (21). A negative control slide was prepared by adding no primary antibody. 

**Statistical Analysis**

Statistical analysis was performed using SPSS software, version 18.0 (SPSS, Inc., Chicago, IL., USA). Quantitative variables are expressed as mean ± SD. Qualitative variables are expressed as numbers (percentages). The data were evaluated with Mann-Whitney Test, Fisher's exact test, or Chi-square.

## Results

**Fig. 1:**
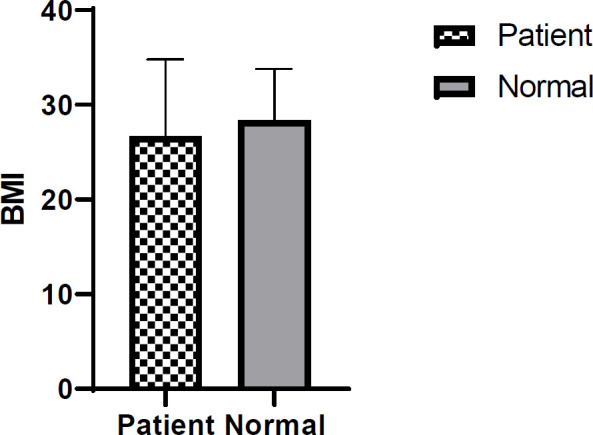
Mean BMI in the two groups

In this research, 90 participants with an age mean of 55.48 ± 12.28 years were enrolled. Normal and CRC groups included 32 (69.56%) and 21 (47.72%) female subjects respectively. As presented in Figure 1, BMI in the patients (26.66±8.11) and controls (28.34±5.44) showed no statistical difference (*P*=0.250). 

The body fat percentage in the two groups is shown in Table 1. While total body fat was higher in normal participants, visceral fat was higher in the patients.

As provided by Table 2, although the mean concentration of leptin was higher in serum samples of the patients (124.47 ng/mL vs. 115.8), no statistically different was found.

Results of Ob-R immunostaining were listed as four categories. Data revealed that 15.9% of CRC tissues were strongly positive while no normal sample was positive (Table 3 and Figure 1).

**Table 1:** The body fat composition

	group	N	Mean	Std. Deviation	p-value
Body fat	Control	46	35.9326	11.97629	0.001
	CRC	44	27.9795	10.79275	
visceral fat	Control	46	8.22	2.882	0.002
	CRC	44	13.37	10.026	

**Table 2:** Serum leptin concentration in the study groups

Group	Leptin concentration (ng/mL)			P-value
Normal (N=46)	Mean Leptin		115.8	0.897
	Percentile	25	87.75	
		50	110.83	
		75	130.34	
CRC (N=44)	Mean Leptin		124.47	
	percentile	25	100.65	
		50	111.8	
		75	130.89	

**Table 3:** Immunostaining of Ob-R in the tissue samples

Positive	Moderately positive	Weakly positive	Negative	
**0**	2(4.3%)	44 (95.7%)	0	**Normal**
**7 (15.9%)**	0	23 (52.3%)	14 (31.8%)	**CRC**
**7**	2	67	14	**Total**

**Fig. 2:**
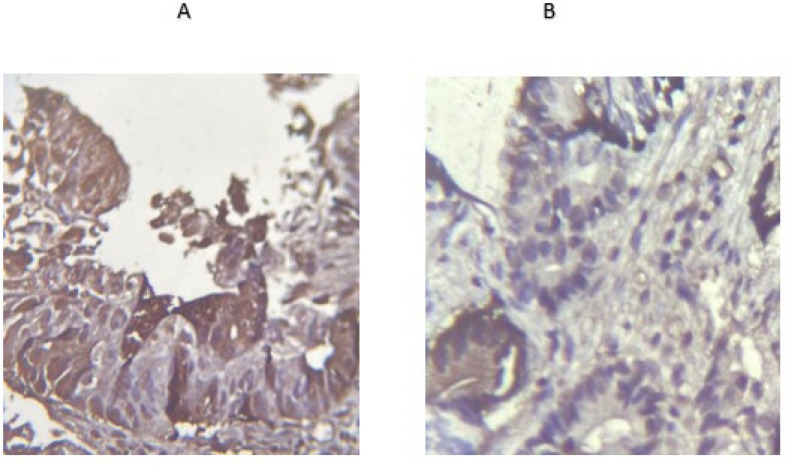
IHC positive (A) and negative (B) samples

By dividing the results into two categories (negative+weak and moderate+strong), CRC cases showed an insignificantly higher Ob-R detection rate (*P*=0.086) which is presented in Figure 2. 

In another analysis, any possible association between serum leptin and anthropometric parameters was investigated. BMI, total, and visceral fat percentage according to the Kendall rank coefficient were not correlated with leptin. 

**Fig. 3:**
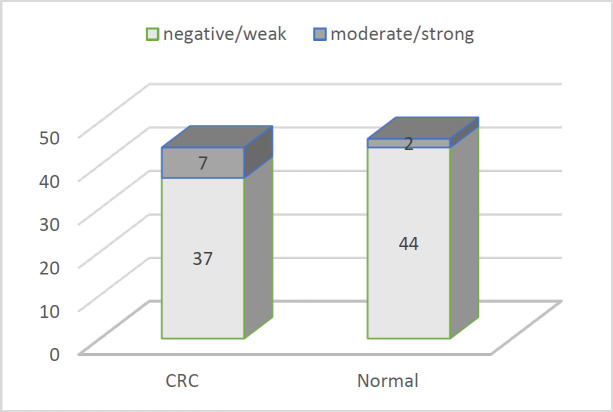
Data on the Ob-R IHC categories

**Table 4:** Correlations between leptin, BMI, and fat percentage

Group			Leptin concentration (ng/ml)
Normal			0.006
	BMI	Correlation Coefficient	
		Sig. (2-tailed)	0.955
	
	Body fat	Correlation Coefficient	-.082
		Sig. (2-tailed)	0.421
	
	visceral fat	Correlation Coefficient	-.008
		Sig. (2-tailed)	0.939
	
CRC	BMI	Correlation Coefficient	-.081
		Sig. (2-tailed)	0.442
	
	Body fat	Correlation Coefficient	-.171
		Sig. (2-tailed)	0.101
	
	visceral fat	Correlation Coefficient	-.080
		Sig. (2-tailed)	0.452
	

## Discussion

Colorectal cancer is one of the leading causes of mortality and morbidity worldwide, thus representing a public health concern. A large body of evidence suggests that substantially increased CRC risk is associated with obesity (10). Leptin is a key regulator of food intake and energy balance. Although mainly produced by adipocytes, leptin is presented in a variety of tissues, including the placenta, stomach, skeletal muscle, and colonic mucosa (22). Some studies indicate leptin as a growth factor for colon cancers cell in vitro (23). The present study investigated serum leptin and tissue Ob-R expression in the samples including cancerous and normal colonic mucosa. Mean leptin levels were not different between the groups also tumor tissues had higher strongly positive Ob-R staining however insignificant. Although it should be noted that fat percentage in both total and visceral forms did not correlate with the serum leptin.

One study in Saudi Arabia investigated the expression of leptin receptors in the tissue samples of colorectal adenoma, cancer, and normal mucosa. High Ob-R protein expression was detected (87.3%) in the examined CRCs. The corresponding values were 58.3% and 5.2% for adenomas and normal specimens, respectively. Ob-R immunohistochemical staining was a favorable prognostic marker and related to early TNM stage and improved survival. Also, no association was reported with BMI and gender (24). 

In contrast, the study of Tutino (25) in Italy revealed patients with higher tumor stages had increased leptin receptor serum levels, although this difference was not statistically significant. Moreover, there was a positive association between advanced tumor stages and leptin/leptin receptor plasma levels. The mentioned study did not include normal subjects and variables such as BMI. Similarly, Vuletic et al, considered Ob-R expression as a poor prognostic factor in CRC as it was positively related to the neoangiogenesis index (26).

In a large European prospective study and different variable matching models, neither leptin nor its receptor serum levels (sOB-R) were associated with rectal and overall CRC cancers. An inverse association between colon cancer and circulating sOB-R and leptin was observed (27).

In a study Kumor *et al.*, Measured serum leptin concentration in a series of colorectal polyps, adenocarcinoma, and normal subjects. Findings demonstrated lower leptin levels in the CRC patients than that in other groups (16). Also, in the study of F Fusun Bolukbas *et al.* (28) patients with gastrointestinal malignancy (gastric and colorectal) had lower levels of the serum leptin than healthy subjects. 

Visceral obesity is noted as a risk factor for developing CRC (29). Also, there are reports introducing it as a prognostic marker in colorectal malignancy (30). In the present study, patients had significantly higher visceral fat. However, total and visceral fat showed no association with leptin. In nondiabetic participants, leptin was a predictor of visceral fat as declared by Sun Ok Song *et al.* (31). In a case-control study on patients with reflux esophagitis no correlation was found between leptin and visceral fat (32), which is similar to the presented findings. Leptin may be more associated with subcutaneous fat than visceral fat especially when adjusted for confounding including activity, age, sex, etc. (33) 

Considering all mentioned results, the role of leptin in the development and prognosis of CRC seems paradoxical, and whether it is a friend or foe, depends on the characteristics studied. Its potential to enhance immune responses highlights the possible benefits of cancer immunotherapy while triggering angiogenesis or epithelial−mesenchymal transition process shows tumor-promoting ability (34). We found no association between leptin and its receptor with CRC. This might be due to population differences, methods applied for the detection of leptin and its receptor, or sample size.

## Limitation

The limitations to declare include the relative small sample size along with lack of investigating genetic variants of leptin and Ob-R in participants which we recommend further studies to consider. 

## Conclusion

None.

## Author Contribution Statement

M, AF; supervised all steps. O, A and L, V conducted assays. V, ON; writing and editing. T, T; I, M; R, S, and A K; were in charge of patient enrollment. R, AN aided in statistical analysis. M, M was responsible for sorting and entering data All authors have approved the final version of the manuscript.

## Acknowledgments

Special thanks to the Deputy of Research and Technology of Mazandaran University of Medical Science for financial support.

## Funding

This study was funded by the Deputy of Research and Technology of Mazandaran University of Medical Sciences.

## Conflict of Interest

None.

## References

[1] Dornbush S, Aeddula NR Physiology, Leptin. [Updated 2022 Apr 14]. In: StatPearls [Internet]. Treasure Island (FL): StatPearls Publishing; 2022 Jan.

[2] Caro JF, Sinha MK, Kolaczynski JW, Zhang PL, Considine RV (1996). Leptin: the tale of an obesity gene. Diabetes..

[3] Zhang Y, Chua Jr S (2011). Leptin function and regulation. Comprehensive physiology..

[4] Tokuyama-Toda R, Satomura K ( 2016). The Physiological Roles of Leptin in Skin Wound Healing. Wound Healing-New insights into Ancient Challenges.

[5] Geary N, Martini L ( 2004). Hunger and Satiation. Encyclopedia of Endocrine Diseases.

[6] Mengie Ayele T, Tilahun Muche Z, Behaile Teklemariam A, Bogale Kassie A, Chekol Abebe E (2022). Role of JAK2/STAT3 Signaling Pathway in the Tumorigenesis, Chemotherapy Resistance, and Treatment of Solid Tumors: A Systemic Review. J Inflamm Res..

[7] Sung H, Ferlay J, Siegel RL, Laversanne M, Soerjomataram I, Jemal A (2021). Global Cancer Statistics 2020: GLOBOCAN Estimates of Incidence and Mortality Worldwide for 36 Cancers in 185 Countries. CA Cancer J Clin..

[8] Maajani K, Khodadost M, Fattahi A, Shahrestanaki E, Pirouzi A, Khalili F (2019). Survival Rate of Colorectal Cancer in Iran: A Systematic Review and Meta-Analysis. Asian Pac J Cancer Prev..

[9] Wong MC, Ding H, Wang J, Chan PS, Huang J (2019). Prevalence and risk factors of colorectal cancer in Asia. Intest Res..

[10] Bahr I, Jaeschke L, Nimptsch K, Janke J, Herrmann P, Kobelt D (2022). Obesity, colorectal cancer and MACC1 expression: A possible novel molecular association. Int J Oncol..

[11] Uddin S, Hussain AR, Khan OS, Al-Kuraya KS (2014). Role of dysregulated expression of leptin and leptin receptors in colorectal carcinogenesis. Tumour Biol..

[12] Haque I, Ghosh A, Acup S, Banerjee S, Dhar K, Ray A (2018). Leptin-induced ER-alpha-positive breast cancer cell viability and migration is mediated by suppressing CCN5-signaling via activating JAK/AKT/STAT-pathway. BMC Cancer..

[13] Aparicio T, Kotelevets L, Tsocas A, Laigneau JP, Sobhani I, Chastre E (2005). Leptin stimulates the proliferation of human colon cancer cells in vitro but does not promote the growth of colon cancer xenografts in nude mice or intestinal tumorigenesis in Apc(Min/+) mice. Gut..

[14] Park KB, Kim EY, Chin H, Yoon DJ, Jun KH (2022). Leptin stimulates migration and invasion and maintains cancer stemlike properties in gastric cancer cells. Oncol Rep..

[15] Fenton JI, Lavigne JA, Perkins SN, Liu H, Chandramouli GV, Shih JH (2008). Microarray analysis reveals that leptin induces autocrine/paracrine cascades to promote survival and proliferation of colon epithelial cells in an Apc genotype-dependent fashion. Mol Carcinog..

[16] Kumor A, Daniel P, Pietruczuk M, Malecka-Panas E (2009). Serum leptin, adiponectin, and resistin concentration in colorectal adenoma and carcinoma (CC) patients. Int J Colorectal Dis..

[17] Meerson A, Yehuda H (2016). Leptin and insulin up-regulate miR-4443 to suppress NCOA1 and TRAF4, and decrease the invasiveness of human colon cancer cells. BMC Cancer..

[18] Endo H, Hosono K, Uchiyama T, Sakai E, Sugiyama M, Takahashi H (2011). Leptin acts as a growth factor for colorectal tumours at stages subsequent to tumour initiation in murine colon carcinogenesis. Gut..

[19] Socol CT, Chira A, Martinez-Sanchez MA, Nunez-Sanchez MA, Maerescu CM, Mierlita D (2022). Leptin Signaling in Obesity and Colorectal Cancer. Int J Mol Sci..

[20] Montazer F, Alizadeh-Navaei R (2019). Expression of GLUT1 in Neoplastic Cells of Papillary Thyroid Cancer. Turkish Journal of Oncology..

[21] Uchiyama T, Takahashi H, Sugiyama M, Sakai E, Endo H, Hosono K (2011). Leptin receptor is involved in STAT3 activation in human colorectal adenoma. Cancer Sci..

[22] Fantuzzi G (2009). Three questions about leptin and immunity. Brain Behav Immun..

[23] Aparicio T, Kotelevets L, Tsocas A, Laigneau J-P, Sobhani I, Chastre E (2005). Leptin stimulates the proliferation of human colon cancer cells in vitro but does not promote the growth of colon cancer xenografts in nude mice or intestinal tumorigenesis in ApcMin/+ mice. Gut..

[24] Uddin S, Bavi PP, Hussain AR, Alsbeih G, Al-Sanea N, Abduljabbar A (2009). Leptin receptor expression in Middle Eastern colorectal cancer and its potential clinical implication. Carcinogenesis..

[25] Tutino V, Notarnicola M, Guerra V, Lorusso D, Caruso MG (2011). Increased soluble leptin receptor levels are associated with advanced tumor stage in colorectal cancer patients. Anticancer Res..

[26] M SV, V SM, S AJ, J TZ, M SK, F CV (2019). Clinical significance of Leptin receptor (LEPR) and Endoglin (CD105) expressions in colorectal adenocarcinoma. Journal of BUON : official journal of the Balkan :union: of Oncology..

[27] Aleksandrova K, Boeing H, Jenab M, Bueno-de-Mesquita HB, Jansen E, van Duijnhoven FJ (2012). Leptin and soluble leptin receptor in risk of colorectal cancer in the European Prospective Investigation into Cancer and Nutrition cohort. Cancer Res..

[28] Bolukbas FF, Kilic H, Bolukbas C, Gumus M, Horoz M, Turhal NS (2004). Serum leptin concentration and advanced gastrointestinal cancers: a case controlled study. BMC Cancer..

[29] Lee JY, Lee HS, Lee DC, Chu SH, Jeon JY, Kim NK (2014). Visceral fat accumulation is associated with colorectal cancer in postmenopausal women. PLoS One..

[30] Park JW, Chang SY, Lim JS, Park SJ, Park JJ, Cheon JH (2022). Impact of Visceral Fat on Survival and Metastasis of Stage III Colorectal Cancer. Gut Liver..

[31] Song SO, Han SJ, Kahn SE, Leonetti DL, Fujimoto WY, Boyko EJ (2021). Leptin and Adiponectin Concentrations Independently Predict Future Accumulation of Visceral Fat in Nondiabetic Japanese Americans. Obesity (Silver Spring)..

[32] Nam SY, Choi IJ, Ryu KH, Park BJ, Kim YW, Kim HB (2015). The effect of abdominal visceral fat, circulating inflammatory cytokines, and leptin levels on reflux esophagitis. J Neurogastroenterol Motil..

[33] Genske F, Kuhn JP, Pietzner M, Homuth G, Rathmann W, Grabe HJ (2018). Abdominal fat deposits determined by magnetic resonance imaging in relation to leptin and vaspin levels as well as insulin resistance in the general adult population. Int J Obes (Lond)..

[34] Jiménez-Cortegana C, López-Saavedra A, Sánchez-Jiménez F, Pérez-Pérez A, Castiñeiras J, Virizuela-Echaburu JA (2021). Leptin, Both Bad and Good Actor in Cancer. Biomolecules..

